# Reliability and validity study of Sino-nasal outcome test 22 (Thai version) in chronic rhinosinusitis

**DOI:** 10.1186/s12901-017-0047-7

**Published:** 2017-12-29

**Authors:** Jate Lumyongsatien, Waralak Yangsakul, Chaweewan Bunnag, Claire Hopkins, Pongsakorn Tantilipikorn

**Affiliations:** 1 Department of Otorhinolaryngology Head and Neck Surgery, Faculty of medicine Siriraj hospital, Mahidol university, 2 Thanon Arun Amarin, Khwaeng Siriraj, Khet Bangkok Noi, Bangkok, 10700 Thailand; 2 DM Guys and St Thomas’ Hospital, ENT Department, Great Maze Pond, London, SE1 9RT UK

**Keywords:** Chronic disease, Language, Quality of life, Reproducibility of results, Sinusitis, Surveys and questionnaires, Translations

## Abstract

**Background:**

Chronic rhinosinusitis (CRS) is one of common health conditions that affects patients’ health-related quality of life. Our purpose is to assess the reliability and validity of Thai-version of Sino-Nasal Outcome Test 22 in chronic rhinosinusitis.

**Methods:**

Permission for translation of SNOT-22 from English language to Thai language was obtained from the developer. The translation process was done based on the international standard of translation method. A total of 80 subjects were recruited into the study and divided into two groups comprising of 50 patients with chronic rhinosinusitis and 30 healthy volunteers. Cronbach’s α and Intraclass correlation coefficient were evaluated for its reliability. Validity test was evaluated against VAS score, SF-36 (Thai version) questionnaire and CT scan (based on Lund-Mackay score). Responsiveness was assessed between pre-operative and post-operative scores in 34 patients.

**Results:**

The Thai version of SNOT-22 showed good reliability according to high value of Cronbach’s α coefficient (*r* = 0.929) and intraclass correlation coefficient (*r* = 0.935). It also showed good validity by its ability to differential the patients with chronic rhinosinusitis from normal (*p* < 0.001), and different severity of symptoms (*p* < 0.05). In addition, the SNOT-22 Thai version also showed good responsiveness when compared between pre-operative and post-operative scores (p < 0.001) and also well-performed in effect size calculation (1.37).

**Conclusion:**

We demonstrated that Thai -version of SNOT-22 has good reliability and validity, suitable for evaluation of chronic rhinosinusitis symptoms together with severity of the disease and response to treatment.

**Trial registration:**

Thai clinical trials registry TCTR20170320003. Date of registration 20/03/2017 (retrospectively registered).

## Background

Chronic rhinosinusitis (CRS) is a common chronic condition affecting significant portion of population. It has been showed that CRS affects 5–15% of the general population both in Europe and the United states [1]. Using general measurement of quality of life (QOL) questionnaire, CRS has been found to affect patient’s QOL not less than other conditions such as congestive heart failure, angina, chronic obstructive lung disease and back pain [2]. Although the general questionnaire, such as SF-36 was demonstrated to be useful in assessment of CRS patient’s QOL, disease-specific questionnaire may be more suitable to evaluate many aspects of the disease [3].

SNOT-20 (Sino-Nasal Outcome test) is one of the widely used disease-specific questionnaire for CRS. It contains 20 questions of CRS-related symptoms/QOL and has been demonstrated for its validity and reliability [4]. However, SNOT −20 lacks 2 important symptoms that commonly found in sinonasal disease i.e. nasal obstruction and loss of sense of smell and taste [5, 6]. SNOT-22 is a modification of SNOT-20 with 2 additional items addressing nasal obstruction and smell/taste problem [6]. The validity and reliability of SNOT-22 was well established [6]. In the present time, SNOT-22 is widely used for evaluation of sinonasal diseases and has been translated and re-validated from original English version into several languages, including Chinese, Portuguese, Greek and French [7–10].

According to its popularity and usefulness, the aim of this study is to translate SNOT-22 into Thai version and make a validation of Thai language questionnaire in Thai CRS patients.

## Methods

**Fig. 1:**
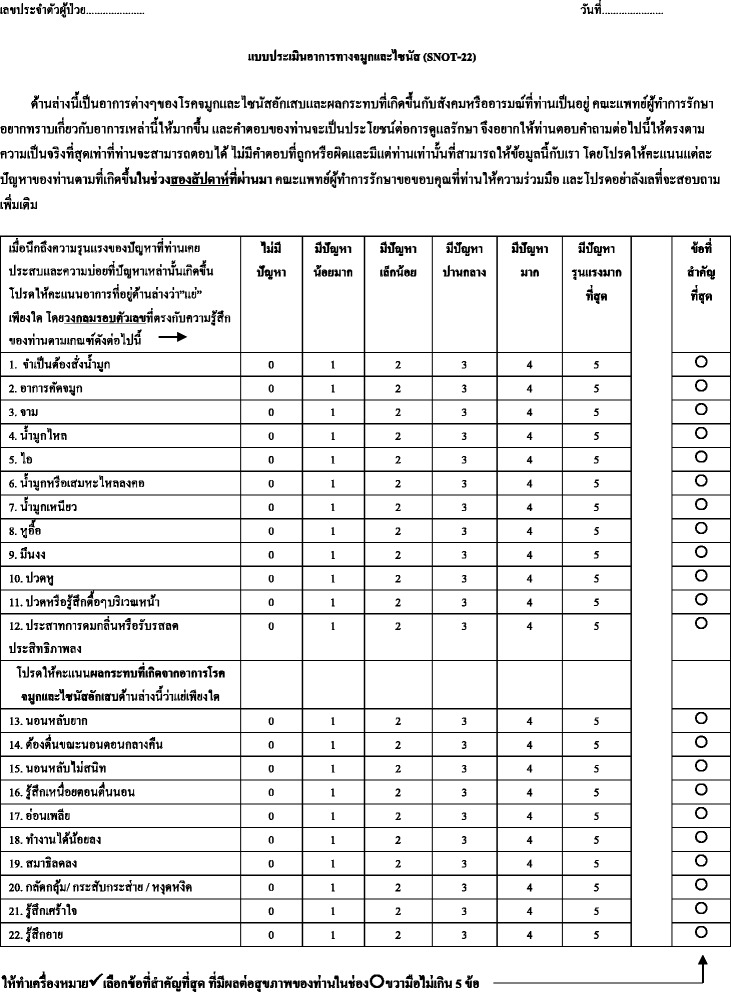
Thai version of SNOT-22

The study was approved by Siriraj institutional review board of Human Research Protection Unit of Faculty of Medicine, Siriraj hospital, Mahidol university. Permission for translation was obtained from the owners of the questionnaire (Piccirillo JF, Hopkins C). Initial translation of SNOT-22 from English to Thai language was made by two Thai native speakers with good academic background in English from Chulalongkorn and Mahidol universities. Backward translation from Thai to English language was performed by an English native speaker with fluency in Thai. Content of Thai-version questionnaire was evaluated to be correct and had the same meaning as in original questionnaire by two rhinologists in our department. Ten volunteers of normal population were collected to check that the questionnaire could be understood (Fig. 1).

All subjects were over 18-year-old and could read and write Thai. Diagnosis of CRS was based on diagnostic criteria of European Position Paper on Rhinosinusitis and Nasal Polyps 2012 [1]. Questionnaire completion was divided into 3 visits. The first visit questionnaire was completed both in CRS and normal group at the first day of enrollment. The second and third visit questionnaire were completed only in CRS group. The second visit questionnaire was completed at 2 weeks after first visit. The third questionnaire, which completed only in CRS patients who underwent sinus surgery, was done 12 weeks after the operation. Pre-operative CT scan of paranasal sinuses and Lund-Mackay score [11] record was done in every operated case. Visual analog scale (VAS) of sino-nasal symptoms and SF-36 score (Thai version) [12] was recorded in all subjects.

Statistical analysis was performed using SPSS program version 18.0 for Windows (SPSS Inc., Chicago, IL, USA). Internal consistency was analyzed by calculating Cronbach’s α. Test-retest reliability was calculated by comparing SNOT-22 between first and second visit (2 weeks interval without change in CRS treatment) using intraclass correlation coefficient. Validity was calculated by comparing SNOT-22 score between CRS and normal group using independent sample T-test. Correlation between SNOT-22 score and SF-36 score/Lund-Mackay score was analyzed by calculating Pearson correlation coefficient. By using VAS, the CRS group was divided into 3 subgroups according to VAS score (mild 0–4, moderate >4–8 and severe >8–10) and SNOT-22 score between 3 subgroups was compared by one-way ANOVA. Responsiveness and sensitivity to change was analyzed by comparing pre/post-operative SNOT-22 score in patients who underwent sinus surgery. Magnitude of treatment effect from surgery was determined by calculating effect size.

## Results

A total of 80 subjects, 30 normal volunteers and 50 CRS patients, were recruited. Subjects’ demographic data was shown in Table 1.

**Table 1:** Demographic data

	Normal	CRS
Total number	30	50
Mean age in years (±SD)	46.43 (±11.138)	51.84(±14.818)
Male	17	24
Female	13	26

Mean score of SNOT-22 in CRS group was 50.36 ± 20.67 and in normal group was 7.70 ± 7.739. Mean score of VAS in CRS group and normal subject were 5.83 ± 2.58 and 0.00 ± 0.00 respectively (Table 2). Mean Lund-Mackay score of CT scan of paranasal sinuses in CRS group was 12.03 ± 6.90. Mean value of SF-36 and Lund-Mackay score in CRS group was shown in Table 3. Internal consistency and test-retest reliability of the questionnaire were analyzed, by calculating Cronbach α and intraclass correlation coefficient respectively. By using independent sample T-test comparing SNOT-22 score between normal and CRS group, Validity of the questionnaire was obtained. The results were shown in Table 4.

**Table 2:** VAS and SNOT-22 score

	Normal	CRS
VAS	0.00 ± 0.00	5.83 ± 2.58
SNOT-22 score	7.70 ± 7.39	50.36 ± 20.67

**Table 3:** SF-36 and Lund-Mackay score of CRS group

	Mean ± SD
SF-36	
Physical function	58.10 ± 21.64
Role physical	46.50 ± 41.35
Bodily pain	54.04 ± 24.53
General health	39.82 ± 16.81
Vitality	53.30 ± 15.31
Social function	59.00 ± 23.39
Role emotional	33.99 ± 38.97
Mental health	60.60 ± 13.93
Lund-Mackay score	12.03 ± 6.90

**Table 4:** Cronbach’s α, Intraclass correlation coefficient and SNOT-22 in normal and diseased groups

	Cronbach’s α	Intraclass correlation coefficient	
SNOT-22 score	0.929	0.935	
	Score in CRS group	Score in normal group	*p*-value
SNOT-22 score	50.36 ± 20.67	7.70 ± 7.39	<0.001

CRS patients were divided into 3 groups, according to VAS (mild 0–4, moderate >4–8 and severe >8). SNOT-22 score between groups were analyzed by one-way ANOVA. The difference of the SNOT-score between groups was statistically significant and was shown in Table 5.

**Table 5:** SNOT-22 score between groups in CRS

		VAS		
	Mild *n* = 8	Moderate *n* = 25	Severe *n* = 17	
SNOT-22 score	37.13 ± 21.59	45.52 ± 19.74	63.71 ± 14.39	Mild vs Moderate group *p* = 0.004 Moderate vs Severe *p* = 0.008

SF-36 and Lund-Mackay score were compared with SNOT-22 score by Pearson correlation test. SF-36 was correlated to SNOT-22 in some domains. There was no correlation between Lund-Mackay and SNOT-22 score (Table 6).

Responsiveness/sensitivity to change was analyzed SNOT-22 score and VAS in 34 patients who underwent surgical treatment using paired-sample T-test. The effect size was calculated from change in SNOT-22 score. The result was shown in Table 7.

**Table 6:** Correlation between SNOT-22 and SF-36/Lund-Mackay score

	SNOT-22	
	*r*	*p*-value
SF-36		
Physical function	−0.372	0.008
Role physical	−0.489	<0.001
Bodily pain	−0.484	<0.001
General health	−0.435	0.002
Vitality	−0.217	0.130
Social function	−0.531	<0.001
Role emotional	−0.321	0.023
Mental health	−0.224	0.118
Lund-Mackay score	0.062	0.727

**Table 7:** Pre/post-operative change in SNOT-22 and VAS

	Pre-operative (mean ± SD)	Post-operative (mean ± SD)	*p*-value	effect size
SNOT-22 score	50.62 ± 20.01	28.97 ± 15.69	<0.001	1.37
VAS	6.24 ± 2.03	3.51 ± 2.47	<0.001	

## Discussion

Chronic Rhinosinusitis is a common chronic disease that has substantial effect on quality of life of the patients. Accurate evaluation of QOL is the crucial part both in treatment and research aspects. There are many kinds of questionnaire that have been used and studied. SNOT-22 is a short, easy to do and validated questionnaire for evaluation of QOL of the CRS patients that recommended to use in literatures [1, 13].

In our study, we demonstrate that Thai-version, as in original English version, of SNOT-22 is a valid and reliable tool for assessment of CRS patients. The questionnaire itself can differentiate CRS patient from normal population (50.36 ± 20.67 vs 7.70 ± 7.39 *p* < 0.001). Moreover, among CRS patients with different severity according to VAS, SNOT-22 score was significantly different between severity groups. This result can be translated that the questionnaire can be used to stratify the severity of the CRS patients. The internal consistency and reliability over time are solid, giving calculated Cronbach’s α and intraclass correlation coefficient 0.929 and 0.935 respectively. In CRS patients who underwent surgery of the paranasal sinuses, SNOT-22 score showed significant reduction at 3 months after surgery (50.62 ± 20.01 vs 28.97 ± 15.69 p < 0.001). This result implies that the questionnaire has very good responsiveness to treatment.

As expected, SNOT-22 Thai version is not correlated well with SF-36 and has no correlation with Lund-Mackay scoring of CT scan of PNS. This result is in the same line with previous study that SF-36 is a general questionnaire about patient’s health status not specific to CRS symptoms and patient’s CRS symptoms are not correlated with severity of the CT scan [10, 14].

There was a study of validity and reliability of Thai-language SNOT-22 published in January 2017 by Numthavaj et al. showing that Thai SNOT-22 is valid and reliable in Thai CRS patients [15]. Even though the results regarding validity and reliability are not different between the previous and the present study, we provide more data which were not demonstrated in the previous research. Those are the data analysis compared SNOT-22 with SF-36/ Lund-Mackay score, SNOT-22 score in normal control compared with CRS patients. Moreover, we also demonstrate that the Thai-language SNOT-22 questionnaire has good responsiveness/sensitivity to change, which has not been analyzed before.

This study has its strength in the terms of the reliability and validity. The discrimination power of SNOT-22 Thai version can be shown statistically by the change of scoring after surgical intervention. Nevertheless, the minimal clinically important difference (MCID) should be further studied in order to determine it discrimination power in the clinical practice. The limitation in our research is we did not study CRS with polyps and CRS without polyps separately. If there was difference between groups, our study results would be changed to some extent.

## Conclusion

SNOT-22 Thai version shows good reliability and validity as its original version. It can be utilized as the validated questionnaire for outcome measurement for CRS. As the SNOT-22 is the most accepted validated questionnaire for CRS, the utilization of SNOT-22 Thai version can be implemented for multi-national research purpose.

## Abbreviations

CRS: Chronic rhinosinusitis
MCID: Minimal clinically important difference
QOL: Quality of life
SF-36: 36-item Short form health survey
SNOT-22: Sino-nasal outcome test 22
VAS: Visual analog scale
